# A brief review of vertebrate sex evolution with a pledge for integrative research: towards ‘*sexomics*’

**DOI:** 10.1098/rstb.2020.0426

**Published:** 2021-08-30

**Authors:** Matthias Stöck, Lukáš Kratochvíl, Heiner Kuhl, Michail Rovatsos, Ben J. Evans, Alexander Suh, Nicole Valenzuela, Frédéric Veyrunes, Qi Zhou, Tony Gamble, Blanche Capel, Manfred Schartl, Yann Guiguen

**Affiliations:** ^1^ Leibniz-Institute of Freshwater Ecology and Inland Fisheries—IGB (Forschungsverbund Berlin), Müggelseedamm 301, 12587 Berlin, Germany; ^2^ Amphibian Research Center, Hiroshima University, Higashi-Hiroshima 739-8526, Japan; ^3^ Department of Ecology, Faculty of Science, Charles University, Viničná 7, 12844 Prague, Czech Republic; ^4^ Department of Biology, McMaster University, Life Sciences Building Room 328, 1280 Main Street West, Hamilton, Ontario, Canada L8S 4K1; ^5^ School of Biological Sciences, University of East Anglia, Norwich Research Park, Norwich NR4 7TU, UK; ^6^ Department of Organismal Biology—Systematic Biology, Evolutionary Biology Centre, Science for Life Laboratory, Uppsala University, Norbyvägen 18D, 75236 Uppsala, Sweden; ^7^ Department of Ecology, Evolution, and Organismal Biology, Iowa State University, Ames, IA 50011, USA; ^8^ Institut des Sciences de l'Evolution de Montpellier, ISEM UMR 5554 (CNRS/Université de Montpellier/IRD/EPHE), Montpellier, France; ^9^ MOE Laboratory of Biosystems Homeostasis and Protection and Zhejiang Provincial Key Laboratory for Cancer Molecular Cell Biology, Life Sciences Institute, Zhejiang University, Hangzhou, Zhejiang 310058, People's Republic of China; ^10^ Department of Neuroscience and Developmental Biology, University of Vienna, A-1090 Vienna, Austria; ^11^ Department of Biological Sciences, Marquette University, Milwaukee, WI 53201, USA; ^12^ Department of Cell Biology, Duke University Medical Center, Durham, NC 27710, USA; ^13^ Developmental Biochemistry, Biocenter, University of Würzburg, 97074 Würzburg, Germany; ^14^ The Xiphophorus Genetic Stock Center, Department of Chemistry and Biochemistry, Texas State University, San Marcos, TX 78666, USA; ^15^ INRAE, LPGP, 35000, Rennes, France

**Keywords:** evolution, genomics, reproduction, vertebrates, sex chromosomes, sex determination

## Abstract

Triggers and biological processes controlling male or female gonadal differentiation vary in vertebrates, with sex determination (SD) governed by environmental factors or simple to complex genetic mechanisms that evolved repeatedly and independently in various groups. Here, we review sex evolution across major clades of vertebrates with information on SD, sexual development and reproductive modes. We offer an up-to-date review of divergence times, species diversity, genomic resources, genome size, occurrence and nature of polyploids, SD systems, sex chromosomes, SD genes, dosage compensation and sex-biased gene expression. Advances in sequencing technologies now enable us to study the evolution of SD at broader evolutionary scales, and we now hope to pursue a *sexomics* integrative research initiative across vertebrates. The vertebrate *sexome* comprises interdisciplinary and integrated information on sexual differentiation, development and reproduction at all biological levels, from genomes, transcriptomes and proteomes, to the organs involved in sexual and sex-specific processes, including gonads, secondary sex organs and those with transcriptional sex-bias. The *sexome* also includes ontogenetic and behavioural aspects of sexual differentiation, including malfunction and impairment of SD, sexual differentiation and fertility. Starting from data generated by high-throughput approaches, we encourage others to contribute expertise to building understanding of the *sexomes* of many key vertebrate species.

This article is part of the theme issue ‘Challenging the paradigm in sex chromosome evolution: empirical and theoretical insights with a focus on vertebrates (Part I)’.

## 1. Introduction

### (a) Towards an integrative understanding of vertebrate sexual differentiation, development and sex determination

In gonochoristic (for this and other terms see Glossary) vertebrates, the genetic and cellular biological processes determining whether an undifferentiated gonad develops towards male or female exhibit great diversity [1,2]. Sex determination (SD) in vertebrates ranges from environmental SD (ESD) to simple or complex genetic systems (genotypic SD (GSD)) that have evolved repeatedly and independently [3–6]. Great plasticity of the developmental processes determining gonads and their initiation during embryogenesis contrasts with the evolutionary conservation of pathways that regulate development of most other tissues and organs [3,7]. In poikilothermic vertebrates, much of the epigenetics and genetics of SD, sex differentiation and sexual development remains poorly understood, and knowledge in homeotherms is mostly restricted to a few models such as humans, mice and chickens [7]. For fishes and amphibians, a diversity of master SD genes defining sex chromosomes was early postulated [8], with some downstream components of the SD networks appearing conserved. Fascinatingly, recent work has illustrated that the molecular control and regulation of SD factors and gonadal differentiation can substantially differ even among closely related groups with indistinguishable gonadal development at the morphological, histological and cellular levels [3,7,9,10].

**Figure 1.**
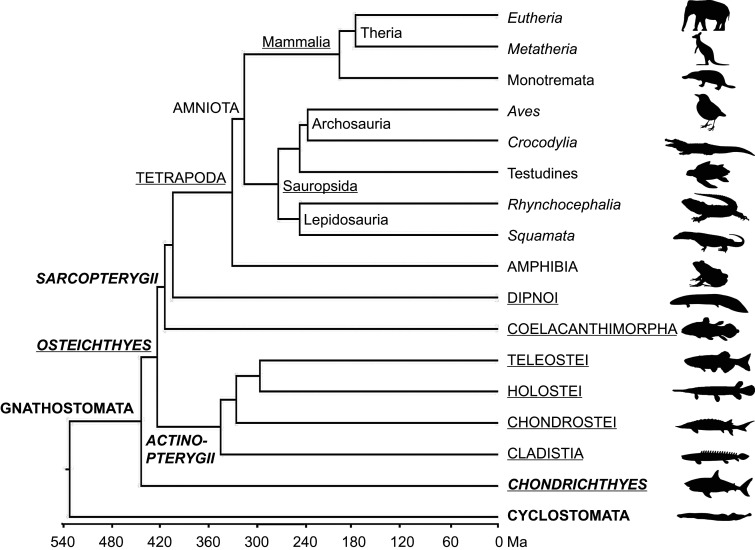
Phylogenetic tree of major clades of vertebrates. Divergence times in millions of years ago (Ma) according to sources provided in the text; typesetting indicates cladistic hierarchies as also used in the text and in table 1.

An interesting heterogeneity exists in the evolution of SD in that some clades exhibit very ancient conservation of sex chromosomes (e.g. birds, therian mammals and many reptile lineages, figure 1), whereas others show frequent evolutionary turnovers with variation even between related clades or even species, such as in many amphibians and fishes, and some reptilian lineages [11]. Highly diverse sex chromosomes may derive from frequent turnovers of SD genes [12,13], suggesting that new SD systems may evolve de novo and independently. Deep homology of some sex chromosome systems across disparate taxa suggest that gene content may predispose certain linkage groups to become sex chromosomes [4,14–16], however, so far with relatively weak support in amniotes [17]. Numerous theoretical concepts and models about transitions among SD systems, degeneration and turnover of sex chromosomes [18–23] often remain to be empirically tested in vertebrates. To understand the diversity of SD and sexual development, a deeper and broader knowledge in multiple species from major phylogenetic lineages is necessary. This may have far-reaching consequences also for other fields, owing to likely coevolution of SD, reproductive modes and life history, which are up to now poorly studied, especially in poikilothermic vertebrates [24–26], although these aspects are very relevant for theoretical and empirical studies of sex ratio ecology and evolution [27].

Here we present an overview of the current knowledge about SD and the genomic resources available for each vertebrate group, as an overture towards a more comprehensive understanding of vertebrate sex evolution. We review the available whole-genome information in all major clades across the vertebrate tree of life, in relation to knowledge about SD, sexual development and reproductive modes, and available genomic resources. We provide an up-to-date overview on divergence times, species numbers, available genomes, genome size, occurrence and nature of polyploids, SD systems, sex chromosomes, SD genes, dosage compensation and sex-biased gene expression.

Despite the fast-developing sequencing technologies allowing genome assemblies of many vertebrates, we consider high-quality genomes only as a starting point that should be complemented by and synthesized with additional information types in order to comprehensively understand sex evolution. We then pledge for an integrative *sexomics* research initiative, which uses high-throughput approaches (e.g. RADSex, PoolSex, RNASex, epigenomics) that would integrate the growing numbers of vertebrate species with an available genome assembly to better understand the evolution of genetic SD and differentiation in vertebrates. This *sexomics* approach could be a starting point for a more in-depth characterization of the ‘complete’ *sexome* of representative species that would require physiological, cell-biological, behavioural information and beyond to better understand sexual reproduction across lineages.

## 2. Overview of current knowledge about sex evolution across the vertebrate phylogeny

### (a) **Vertebrate sister groups: CEPHALOCHORDATA (LANCELETS) and TUNICATA (TUNICATES)**

Extant fish-like lancelets (also called amphioxi; [28,29] are considered the sister group to tunicates and vertebrates (e.g. [30]). Lancelets are gonochorists, but little is known about their SD. Recent genetic evidence suggests a female-heterogametic (ZZ/ZW) GSD system [31]. The karyotype of lancelets is considered to resemble that of ancestral vertebrates [32]. According to traditional models, the early vertebrate ancestors experienced two successive rounds of whole-genome duplications (assigned as 1R, 2R) between approximately 500 and 450 Ma [33,34]. However, Simakov *et al*. [29] suggested three duplication events—the first before the diversification of extant chordates, the second in the ancestor of lampreys, and the third in the ancestor of jawed vertebrates.

Tunicates, the putative sister group of vertebrates, possess a wide array of reproductive systems. Sedentary ascidians are mostly sequential hermaphrodites, but some produce sperms and eggs simultaneously with incompatible cell-surface proteins, preventing self-fertilization [35]. Colonial species reproduce asexually by budding. Appendicularian tunicates are mostly sequential hermaphrodites [36] but the pelagic tunicate *Oikopleura dioica* has an XX/XY genetic sex-determining system with possible dosage compensation [37]. Pyrosomes are hermaphroditic as well, reproducing both asexually and sexually with internal fertilization. Thaliaceans (salps) have complex life cycles, obligatorily alternating between sexual and asexual reproduction, allowing rapid population growth while preserving genetic variability [38]. The oozooid develops from a zygote produced by budding, resulting in a chain of individuals that contains an ovary and a testis. The eggs are fertilized internally and the embryo is brooded by the ‘mother’. The life cycle of doliolids is the most complex, again including asexual reproduction with a sequential hermaphroditic phase (for overview: [39]).

With respect to the chordate ancestor, extant lancelets and tunicates may have a derived sexual development, life cycles and SD systems, which evolved during the hundreds of millions of years of divergence from the vertebrate lineage. Nevertheless, as the closest living vertebrate outgroups, they might provide important insights into the deep evolutionary history of sex-related traits and SD genes in vertebrates.

### (b) **VERTEBRATA (VERTEBRATES)**

#### CYCLOSTOMATA (JAWLESS FISHES)

The branch of jawless vertebrates with its approximately 120 living species branched off approximately 540 Ma from the lineage leading to all other vertebrates during the Cambrian (figure 1). Agnatha comprises the extant clades of hagfishes (Myxini) and lampreys (Petromyzontiformes). Four lamprey genomes are available [40–42], and an assembly from hagfish (Myxinidae) is available. Hagfish genome size (c-value: 2.4–4.5 Gb; [43]) exceeds that of lampreys (1.4–2.4 Gb). Hagfishes and lampreys are oviparous (table 1). SD and sexual development of hagfishes is poorly understood. Some species appear as protogynous hermaphrodites [44], but no functional simultaneous hermaphrodites have been documented [45,55], other species are gonochoristic [56]. It is possible that SD in lampreys is epigenetic/environmental [57]. However, the critical sex differentiation period is unexplored, and the evidence for ESD in lampreys remains equivocal since GSD as a possible alternative has been proposed recently [58]. The sea lamprey genome contains several hundred genes that are eliminated from somatic cells during early development [41]. Other lampreys and hagfish likewise undergo genome elimination [46], but it remains unknown whether genome elimination plays a role in sexual development.

**Table 1.** Overview on available genome assemblies of vertebrates ([42], as December 2020) contrasted with generally known information on sexual phenotype, sex determination mode (SD mode) in gonochorists, system of genotypic sex determination (GSD), reproductive mode, reproduction and master sex determination genes (in GSD species). (Total numbers of species and families were derived from the NCBI taxonomy database and may contain higher than expected numbers owing to the presence of extinct species/families. Herein: x, trait is present; NRSF, trait ‘not reported so far’ despite the availability of at least some data on this topic; no data, to our knowledge, this topic has not been studied/examined; ?, questionable/equivocal evidence.)

vertebrate genome assemblies at NCBI as of December 2020:			sexual phenotype		SD mode in gonochorists		system of GSD			reproductive mode		reproduction					master SD gene	comments
	families	species	gonochorism	hermaphroditism	ESD	GSD	male heterogamety	female heterogamety	multilocus sex det.	oviparous	viviparous	bisexual	parthenogenesis, facultative	parthenogenesis, obligate	gynogenesis	hybridogenesis		
**CYCLOSTOMATA**	**2** (of 3)	**5** (of 546)	x	?	no data	no data	no data	no data	no data	x	x	x	no data	NRSF	no data	no data	no data	hermaphroditism in hagfish questionable [44,45]; genome elimination in both lampreys and hagfishes [46]
**GNATHOSTOMATA**	**487** (of 1061)	**1646** (of 78 773)																
***CHONDRICHTHYES***	**8** (of 56)	**10** (of 1736)	x	NRSF	no data	x	x	?	no data	x	x	x	x	NRSF	NRSF	NRSF	no data	female heterogamety in a stingray questionable; cases of hermaphroditism report rather on intersexuality (rudimentary hermaphroditism) [47]
***OSTEICHTHYES***	**479** (of 1005)	**1636** (of 77039)																
***ACTINOPTERYGII***	**137** (of 498)	**631** (of 38854)																
CLADISTIA	**1** (of 1)	**1** (of 16)	x	no data	no data	no data	no data	no data	no data	x	NRSF	x	no data	no data	no data	no data	no data	
CHONDROSTEI	**1** (of 2)	**2** (of 62)	x	?	NRSF	x	?	x	NRSF	x	NRSF	x	NRSF	NRSF	NRSF	NRSF	no data	questionable (male or female heterogamety) SD reported in *Polyodon* [48,49]
HOLOSTEI	**1** (of 2)	**1** (of 14)	x	no data	no data	no data	no data	no data	no data	x	NRSF	x	no data	no data	no data	no data	no data	
TELEOSTEI	**134** (of 493)	**627** (of 38659)	x	x	x	x	x	x	x	x	x	x	NRSF	NRSF	x	x	multiple	
***SARCOPTERYGII***	**342** (of 507)	**1005** (of 38 185)																
COELACANTHIMORPHA	**1** (of 1)	**1** (of 2)	x	no data	no data	no data	no data	no data	no data	NRSF	x	x	no data	no data	no data	no data	no data	
DIPNOI	**0** (of 3)	**0** (of 17)	x	no data	no data	no data	no data	no data	no data	x	NRSF	x	no data	no data	no data	no data	no data	
TETRAPODA	**341** (of 503)	**1004** (of 38 168)																
AMPHIBIA	**13** (of 71)	**19** (of 10 180)	x	?	?	x	x	x	x	x	x	x	NRSF	NRSF	x (see comment)	x	*Dmrt1?, Dm-W* (some *Xenopus)*, other suggested	*Ambystoma* salamanders reproduce by kleptogenesis [50], previously reported as ‘gynogenesis’ [51]
AMNIOTA	**328** (of 432)	**985** (of 27 990)	x		x	x	x	x		x	x	x	x				* *	
Sauropsida	**206** (of 274)	**564** (of 19 731)																
Archosauria	**180** (of 197)	**506** (of 10 173)																
*Aves*	**177** (of 194)	**502** (of 10 137)	x	NRSF	NRSF	x	NRSF	x	NRSF	x	NRSF	x	?	NRSF	NRSF	NRSF	*Dmrt1*	see main text on parthenogenetic development in domestic birds and [52,53]
*Crocodylia*	**3** (of 3)	**4** (of 36)	x	NRSF	x	NRSF	NRSF	M	NRSF	x	NRSF	x	NRSF	NRSF	NRSF	NRSF	NRSF	
Testudines	**13** (of 14)	**22** (of 366)	x	NRSF	x	x	x	x	NRSF	x	NRSF	x	NRSF	NRSF	NRSF	?	no in ESD/unknown in GSD	see text on *Platemys* and [54]
Lepidosauria	**13** (of 61)	**36** (of 9110)																
*Squamata*	**12** (of 60)	**35** (of 9109)	x	NRSF	x	x	x	x	NRSF	x	x	x	x	x	NRSF	NRSF	no in ESD/unknown in GSD	
*Rhynchocephalia/Sphenodontia*	**1** (of 1)	**1** (of 1)	x	NRSF	x	NRSF	NRSF	NRSF	NRSF	x	NRSF	x	NRSF	NRSF	NRSF	NRSF	NRSF	
Mammalia	**122** (of 158)	**421** (of 8259)																
Monotremata	**2** (of 2)	**2** (of 7)	x	NRSF	NRSF	x	x	NRSF	NRSF	x	NRSF	x	NRSF	NRSF	NRSF	NRSF	*Amh?*	
Theria	**120** (156)	**419** (of 8247)																
*Metatheria*	**8** (of 19)	**8** (of 422)	x	NRSF	NRSF	x	x	NRSF	NRSF	NRSF	x	x	NRSF	NRSF	NRSF	NRSF	*Sry*	
*Eutheria*	**112** (of 137)	**411** (of 7825)	x	NRSF	NRSF	x	x	NRSF	x	NRSF	x	x	NRSF	NRSF	NRSF	NRSF	*Sry*	

#### **GNATHOSTOMATA (JAWED VERTEBRATES)** ***CHONDRICHTHYES (CARTILAGINOUS FISHES)***

With approximately 1200 extant species, cartilaginous fishes comprise the sister group to all other living jawed vertebrates, with elasmobranchs (sharks, rays and relatives) and holocephalans (chimaeras) sharing an Ordovician common ancestor with Osteichthyes approximately 450 Ma [59]. The genomes of only a few species have been characterized, hindered by large genome sizes (2.6–16.6 Gb; [43]). Currently, six sharks [60], two skates [61] and two chimaera genomes [62] have been assembled (recent overview: table 1). The modes of reproduction are very diverse, including yolk-sac viviparity, histotrophy (nutrition of an embryo by uterine secretions), oophagy and placental reproduction [45,63]. Several studies report cases of occasional (facultative) parthenogenetic reproduction giving rise to all-female offspring [64]. Intersexes (often reported as hermaphrodites) were reported in more than 30 elasmobranch species. They frequently showed improper development or maturation rendering one or both sexes nonfunctional [47]. Nevertheless, no functional hermaphroditism was described in this group. SD in Chondrichthyes appears to be largely genotypic with cytogenetic data suggesting XX/XY sex chromosomes in the few studied species of sharks [65] and rays [66], or possibly other forms of male heterogamety in freshwater stingrays (*Potamotrygon*; [67,68]). A ZZ/ZW system was tentatively reported in the stingray *Hypanus americana* [65]. We can conclude that there is currently more information about the evolution of the male genitalia, the claspers [69], than on genetic or possible environmental triggers of SD.

#### ***OSTEICHTHYES (BONY FISHES)***

***ACTINOPTERYGII (RAY-FINNED FISHES)***

With some more than 31 000 species, the ray-finned fishes are a very diverse vertebrate class, largely comprising extant Teleostei and few non-teleosts: Cladistia (bichirs), Chondrostei (sturgeons and paddlefish, and Holostei (bowfins and gars). The ancestor of Teleostei underwent another round of whole-genome duplication (traditionally called ‘3R WGD’, or ‘teleost-specific WGD’; [33,70]).

CLADISTIA (BICHIRS)

These ray-finned fishes diverged more than 350 Ma in the Devonian from other actinopterygians [71]. Cladistia comprise 13 *Polypterus* and a single *Erpetoichthys* species, with large genomes (4.6 Gb to possibly 7.00 Gb) in *Polypterus*, and a similar genome size (4.4 Gb) in *Erpetoichthys* [43]. A BAC-library of the Senegal bichir, *Polypterus senegalus,* was prepared [72]. For the reedfish, *Erpetoichthys calabaricus*, a chromosome-scale genome assembly is available (table 1). Bichirs are egg-laying and share holoblastic embryonic cleavage with sturgeons. Heteromorphic sex chromosomes have not been found in bichirs [73–75]. Given their phylogenetic position, information on SD and development might provide important insights into the ancestral condition of Actinopterygii.

CHONDROSTEI (STURGEONS and PADDLEFISH)

Sturgeons and paddlefish comprise 27 living species [76] that diverged 330 Ma [77] from the ancestor of the Teleostei and Holostei [33,70]. After their divergence from the other ray-finned fish lineages, sturgeons and paddlefish experienced several polyploidization events, yielding extant species karyotypes from basal approximately 120 up to as many as about 380 chromosomes [78] (and even more in single individuals), and moderate to large genome sizes from 1.4 to 4.4 Gb [43]. Several projects are underway to assemble high-quality sturgeon genomes [77], and a paddlefish genome has been recently published [79]. In contrast to other polyploid fishes, sturgeon genomes maintain a high proportion of ohnologues, i.e. they exhibit a slow deduplication process and loss of several homeologous chromosomes (segmental rediploidization), posing major challenges for genome assembly [77,80]. Chondrostei have exclusively oviparous reproduction [76] and share holoblastic cleavage with most amphibians but not teleosts [81]. Sturgeons do not possess cytologically differentiated sex chromosomes [77,82]. The sex ratio of offspring from experimental gynogenesis yielded contradictory results suggesting either male (XX/XY; [48]) or female heterogamety (ZZ/ZW; [49]) in a paddlefish (*Polyodon spathula*) and a female-heterogametic (ZZ/ZW) SD system in sturgeon [83], yet a sex-linked marker was not found for decades [77]. Using chromosome-scale assemblies and pool-sequencing, an approximately 16 kb female-specific sequence from sterlet (*Acipenser ruthenus*) was detected by Kuhl *et al*. [84]. A polymerase chain reaction-genotyping test, yielding female-specific products in six sturgeon species, spanning the entire phylogeny with the most divergent extant lineages (*Acipenser sturio, Acipenser oxyrinchus* versus *Acipenser ruthenus, Huso huso*), stemming from an ancient tetraploidization. Similar results were obtained in two octoploid species (*Acipenser gueldenstaedtii, Acipenser baerii*). Phylogenetic conservation during 180 Myr of sturgeon evolution and across at least one polyploidization event revealed the oldest known vertebrate system with undifferentiated sex chromosomes, based presumably on a ZZ/ZW-mode of sex determination [84].

HOLOSTEI (BOWFINS and GARS)

A single species of extant bowfin (*Amia calva*) [85] from North America and closely related gars (Lepisosteiformes), occurring in North and Central America plus the Caribbean, with seven living species [86] represent the sister taxon of teleosts. These two lineages diverged in the Early Permian (approx. 300 Ma; [71]), before the teleost-specific WGD. Eased by reasonable genome sizes in bowfin (1.0–1.3 Gb) and gars (1.0–1.3 Gb), a gar [87], and most recently the bowfin genome [88] have been assembled. Gars and bowfins are oviparous [89,90] and show holoblastic embryonal cleavage. No information on the SD in Holostei is available and no sex-specific genome regions have been identified so far [88].

TELEOSTEI (TELEOSTS)

The rise of teleosts, which comprise approximately 31 000 species [91]) and thus make up over 99% of all ray-finned fishes (Actinopterygii), was accompanied by the teleost-specific WGD (traditionally assigned as ‘3R WGD’) in their common ancestor approximately 300 Ma [34,71]. Some lineages, e.g. salmonids and carps, independently experienced yet additional WGD events. Teleosts evolved meroblastic embryonal cleavage [92]. To date several hundreds of teleost genomes have been assembled (table 1). Owing to advanced deduplication and diploidization of genomes and relatively small to large genome sizes (0.4–5.3 Gb; with most genomes less than 2.0 Gb; [43], whole-genome sequencing (WGS) of teleosts shows great progress among vertebrates. Teleosts feature the largest diversity of reproductive modes [93]. All-female sperm-dependent parthenogenetic (gynogenetic) or hybridogenetic species of hybrid origin [94,95], and even sequential (protandrous, protogynous or serial, i.e. bidirectional) hermaphroditism [96], in some cases involving socially controlled sex change [97,98] and simultaneous hermaphroditism exist [99,100], the latter including the only self-fertilizing vertebrate [101]. Sexual development of teleosts is also very plastic [102], and sex reversal can be easily induced by hormonal and sometimes by environmental triggers or treatments [103], rendering them susceptible to endocrine-disruptive pollution [104]. Data in zebrafish and medaka indicate that germ cell number can drive SD [105,106].

Teleosts show the widest variety of sex-determining mechanisms among vertebrates [107]. This includes gonochorism with ESD and GSD (as well as its environmental modulation), GSD ranging from homomorphic to heteromorphic female (ZZ/ZW) or male heterogametic (XX/XY) systems, plus polygenic SD [108,109] or multiple sex chromosomes [110], with different systems evolved in closely related species. Pure temperature-dependent sex determination (TSD, e.g. [111]) appears to occur in teleosts relatively rarely [107]. In teleosts, the largest number of master SD genes in vertebrates has been characterized [112]. Teleost master SD genes evolved from well-known members of the sexual development regulatory network (the ‘usual suspects’, [3]), stemming in some cases from transcription factors (*Dmrt1*, *Sox3*), *Tgf-beta* signalling pathway members (*Amh, Amhr2, Gsdf, Gdf6*), or exceptionally from an immune gene in salmonids (*Irf9*; [113]), triggering male gonadogenesis through an unknown mechanism. The non-recombining region of young teleost sex chromosomes may be remarkably small, e.g. 300 kb in Atlantic herring (*Clupea harengus*; [114]), some sex chromosomes may even freely recombine, and rarely, the X and Y may differ by just a single nucleotide polymorphism, as reported in the Japanese pufferfish, *Takifugu rubripes* [115]. In teleosts, the research on rewiring of SD- and sex differentiation gene networks is the most advanced [3]. Compared to the huge teleost biodiversity, the discovery of novel SD genes and systems can be expected from WGS of additional species. Many teleosts, among them sequential or simultaneous hermaphrodites and recent polyploids with specific reproductive modes, such as gynogenesis or hybridogenesis [116], still lack the characterization of their genomes, SD systems and SD genes as well as interactions of allospecific sex chromosomes in taxa of hybrid origin.

### 

***SARCOPTERYGII (LOBE-FINNED FISHES)***

Coelacanths and lungfishes are the only living sarcopterygian fishes [117] that all trace back to a divergence in Silurian times, i.e. more than 420 Ma [71]; all other extant sarcopterygians comprise tetrapods.

#### COELACANTHIMORPHA (COELACANTHS)

There are two coelacanth species from southeastern Africa and Sulawesi [118]. Coelacanths are ovoviviparous [119,120]. A coelacanth 2.86 Gb genome of *Latimeria chalumnae* has been assembled [121]. Over 50 genes involved in sex differentiation and gametogenesis were sequenced in *L. chalumnae* and *Latimeria menadoensis,* but no master SD genes have been characterized [122,123]. This situation may not change, given the secretive deep-sea lifestyle of these species and their conservation status (CITES).

#### DIPNOI (LUNGFISHES)

The six living known species of lungfish occur in Africa, South America and Australia. As their closest living relatives, lungfishes are in a uniquely informative phylogenetic position to infer the ancestral condition of tetrapods [124]. Lungfishes are oviparous [125] and show a pattern similar to holoblastic cleavage [92,125]. While coelacanths have moderate vertebrate genome sizes (2.6 Gb; [121]), lungfish genomes range among the largest in vertebrates (49–60 Gb; [43]). Despite their huge size, the assemblies without information about SD systems from the Australian lungfish (*Neoceratodus forsteri*) and the African lungfish (*Protopterus annectens*) have recently been obtained [126,127]. In *P. annectens*, more than 50 genes related to sex differentiation and gametogenesis have been characterized [123]. Master SD genes have not been identified in lungfishes. The availability of captive breeding in some lungfish species might ease elucidation of SD.

#### TETRAPODA (TETRAPODS)

AMPHIBIA (AMPHIBIANS)

Soon after their Devonian divergence (335 Ma; [128]) from Amniota, the amphibian lineage to Gymnophiona (caecilians) branched off from that of Anura (frogs and toads) and Urodela (= Caudata: newts and salamanders), while the latter two clades (Anura, Caudata) diverged in the Early Permian (300 Ma, [129]). Many amphibian families are deeply (100–150 Ma) diverged [130,131], with recent evidence that 88% of anurans (Hyloidea, Microhylidae, Natatanura) underwent a rapid Cretaceous–Palaeogene boundary diversification [132]. Gymnophiona also exhibit deep divergences, raising expectations for major genomic evolutionary differences [133]. Cleavage in most frog and salamander embryos is radially symmetrical and holoblastic. The limited knowledge on caecilians, however, suggests meroblastic cleavage in this group [134].

Although there are more species of amphibians (over 8260; [135]) than mammals (6485; [136,137], to date only 19 amphibian genomes of various quality have been assembled, including 15 out of 7291 Anura, 1 out of 760 of Urodela (Caudata) and 3 out of 213 Gymnophiona [42,135]. Even fewer assemblies have reached chromosome-scale quality. The so far slow progress in amphibian genomics is mostly caused by large genome sizes, reaching from 3.9 to 9.8 Gb in Gymnophiona, 1.9–13.1 Gb in Anura, and huge 16.6–78.2 Gb in Urodela [138], and by the large proportions of repetitive sequences. The ongoing dawn of amphibian genomics will be much enlightened by long-read and three-dimensional technologies [139], with many amphibian families still awaiting their first WGS.

Anurans evolved a great diversity of reproductive modes, with terrestrial eggs and exotrophic aquatic larvae, preceding the frequent and repeated evolutionary rise of direct development (terrestrial eggs, no tadpoles), while non-feeding (endotrophic) larvae never led to direct developers [140]. Newts and salamanders exhibit aquatic larvae (rarely involving exceptional or even obligate neoteny, i.e. larval reproduction), as well as terrestrial eggs, and ovo-viviparity with birth of larvae or fully metamorphosed offspring, rarely boosting development by intrauterine cannibalism [141]. Gymnophiona are oviparous or viviparous [142,143], including rare direct developers [144].

While true parthenogenesis most likely did not evolve in amphibians (table 1), hybridogenetic systems, including male- or female-biased and probably GSD-governed population systems occur in anurans [145] as well as kleptogenesis (previously called ‘gynogenesis’; [51]) in salamanders [50], where all-female hybrids of five ploidy levels acquire full or partial genomes from allospecific males and ‘purge’ genomes from deleterious alleles. Recent auto- and allo- (i.e. hybrid origin) polyploids, presenting in amphibians the highest frequency of all vertebrates, are known from several families of anurans and salamanders [146] but are so far unknown in Gymnophiona. Occasional reports on natural sex change in adult anurans (e.g. [147] in *Hyperolius viridiflavus*) require further examination.

About 96% of the amphibians exhibit undifferentiated sex chromosomes [148,149]. All studied amphibians show GSD and either male (XY/XX) or female (ZZ/ZW) heterogamety [150–152], in addition a putative case of a female W0/00 male SD system [153] and several cases of multiple sex chromosomes [154] have been reported, which form a ring during meiosis in the smoky jungle frog, *Leptodactylus pentadactylus* [155]. While the vast majority of amphibians exhibit homomorphic XX/XY or ZZ/ZW sex chromosome systems, there are several prominent examples of cytogenetically differentiated sex chromosomes [156,157], and for the African bullfrog, *Pyxicephalus adspersus*, a draft genome is pre-published [158], from which potential upregulation of the heterogametic W-chromosome and/or repression in the homogametic Z might inform about dosage compensation. In the cytologically indistinguishable sex chromosomes of the western clawed frog, *Xenopus tropicalis*, male-biased expression of sex-linked transcripts is suspected to be owing to degeneration of the non-recombining portion of the W-chromosome, coupled with incomplete or absent dosage compensation [159]. Cases of sex chromosome-autosome translocations have been shown by cytogenetics [160]. A balanced lethal system in newts (*Triturus*) may have evolved from a vestigial sex chromosome pair [161,162]. Sex chromosomes of most newts and salamanders are homomorphic [157,163], and the observation of balanced sex ratios from clutches is interpreted as indication for GSD but has remained without genetic evidence [164]. Whole-genome approaches in multiple individuals identified the homomorphic sex chromosome of axolotl (*Ambystoma mexicanum*), and a putative approximately 300 kb SD region on the W-chromosome [164,165]. Genomic approaches recently also suggested sex-linked loci in ancient clades of giant salamanders (family Cryptobranchidae; [166,167]). Transcriptomic approaches try to circumvent limitations of huge urodelean genome sizes to address sexual developmental aspects [168,169]. Evidence for heteromorphic sex chromosomes exists for at least one species of Gymnophiona [170].

Homomorphic sex chromosomes in amphibians may be caused by high turnover rates [171], where autosomes evolve into new sex chromosomes [8], as documented in ranid frogs [15] and pipid frogs [10]. Another hypothesis to explain homomorphy is occasional X-Y recombination (‘fountain-of-youth’-model; [9]), assuming recombination arrest in males to be controlled by maleness (i.e. by the sexual phenotype rather than the sex chromosomal genotype). Thus, Y chromosomes may recombine, for example, in sex-reversed XY-females, preventing long-term Y degeneration, supported by data from tree frogs [172], true frogs [173] and Palaearctic green toads [174]. Generally, sex reversal in early developmental stages owing to environmental cues is possible, making semi-aquatic amphibians, like fishes, vulnerable to pollution of aquatic ecosystems with endocrine-disruptive compounds [104,175].

Early studies on SD involved experimental sex reversal [176,177], cytogenetics and crossing experiments [148,149]. In-depth molecular studies on amphibian SD stem mostly from clawed frogs (*Xenopus*), where LG7 is sex-linked in diploid *X. tropicalis* [178,179] and coexisting X, Y and W-chromosomes are suggested [154,159] but no master SD gene is known [180]. The only well-characterized anuran master SD gene is a *Dmrt1*-paralogue, the W-linked *Dm-w* of *Xenopus laevis* [151,181], present in some closely related *Xenopus* species but not in the entire pipid radiation [10,182]. *Dm-w* arose after (and perhaps in response to) tetraploidization [182–184] and may initially not have governed sexual differentiation. *Dmrt1* itself is considered a candidate master SD gene in some hylid frogs [185,186], bufonid toads [174] and common frogs (*Rana temporaria*; [173]), and is also sex-linked in several other ranids [15]. The male versus female-determining molecular mechanisms suggest that parallel amino acid substitutions contributed to the establishment of *Dmrt1Y* (medaka fish) and *Dm-w* (*Xenopus*) as SD genes [187]. A well-studied ranid frog system is that of the Japanese wrinkled frog, *Glandirana rugosa,* with five genetic lineages: the west Japan, east Japan and XY-groups possess XX/XY systems; the ZW- and Neo-ZW groups ZZ/ZW SD systems [188]. In all lineages, the genes *androgen receptor* (*Ar*)*, splicing factor 1* (*Sf-1*) and *Sry-box transcription factor 3* (*Sox3*) are located on the Z and W or X and Y chromosomes [189,190]. In most amphibians, the characterization of diploid and polyploid SD systems, evolution by hybridization and introgression and generally the characterization of SD systems, sex chromosomes and their evolution remain unknown from a genomic perspective.

### 

AMNIOTA (AMNIOTES) Sauropsida (sauropsids, reptiles and birds)

Lepidosauria (lepidosaurs)

*Rhynchocephalia/Sphenodontia (tuatara)* The only extant species in the reptilian order Rhynchocephalia (Sphenodontia), diverged approximately 250 Ma from their sister taxon Squamata (lizards and snakes), is the tuatara (*Sphenodon punctatus*), endemic to New Zealand. The 5 Gb tuatara genome has been recently reported [191], including a list of sex developmental genes [192]. The oviparous tuataras exhibit a unique form of TSD, with females produced below, and males above 22°C [193]. Tuataras possess no sex chromosomes with neither population genomic resources nor global CG-methylation patterns revealing sex specificity [191,193]. Orthologues of genes acting antagonistically in masculinizing (e.g. *Sf1*, *Sox9*) or feminizing (e.g. ​*Rspo1, Wnt4*) networks promoting testicular or ovarian development, have been identified, as were genes implicated in TSD (e.g. *Cirbp*; [7]). This example shows that WGS alone can be insufficient to understand SD, particularly TSD. However, a high-quality genome is an important resource for the evaluation of embryonic transcriptomes or proteomes, which are critical data sources for characterization of genes related to sexual development.

*Squamata (squamates, lizards and snakes)* Squamates, comprising currently more than 11 000 species [194], diverged approximately 250 Ma from Rhynchocephalia [195], while lepidosaurs diverged 277 Ma from archosaurs and turtles [191]. To date, more than 35 genomes have been assembled (table 1). Genome studies are eased by moderate genome sizes, ranging from 1.3 to 3.7 Gb [196]. Squamate reptiles are oviparous or viviparous; ‘ovo-viviparity’ may be difficult to distinguish from viviparity [197]. They mostly exhibit gonochorism, and very rarely true parthenogenesis (females give birth to genetically identical—‘clonal’—daughters; [94,198]). In several clades of lizards and in a blind snake, diploid or triploid all-female obligate parthenogenetic complexes, mostly of hybrid origin, are known [199,200] or arose in the laboratory [201]. Interestingly, natural polyploid reptiles appear only fertile as triploids [202]. Squamates exhibit GSD with male (XX/XY) or female (ZZ/ZW) heterogamety, having undifferentiated or often differentiated, heteromorphic sex chromosomes, or ESD, mostly in the form of TSD [6,18,203–205]. ESD seems relatively rare, currently estimated to occur in roughly 5% of non-avian reptile species [206]. Multiple neo-sex chromosomes evolved via sex chromosome-autosome fusions more frequently in iguanas with male heterogamety than in snakes with female heterogamety [207], which agrees with similar apparent patterns in other vertebrates [110,207,208]. Neither simultaneous nor sequential hermaphrodite species are known in reptiles [209]. Facultative parthenogenesis is well documented in many snake and lizard lineages, with all-female progeny under male heterogamety but all-male progeny under female heterogamety with degenerated W-chromosomes [210]. Facultative parthenogenesis yielding genetically variable offspring of both sexes was discovered in a xantusiid lizard [211]. Five squamate clades (iguanas, lacertid lizards, varanids, skinks and caenophidian snakes) covering approximately 60% of extant squamates show evolutionary conserved sex chromosomes [206,212–216], while other lineages, particularly Acrodonta (agamid lizards and chameleons), boas and pythons, and geckos exhibit more variable SD [18,205,217–219]. In two snake families and the Komodo dragon (*Varanus komodoensis*) with female heterogamety, substantial W-chromosome degeneration and the absence of global Z-chromosome dosage compensation has been shown, dosage balance is largely lacking in Z-specific genes in these species [215,220,221]. By contrast, X-linked genes are twofold upregulated in males and thus fully dosage-compensated in *Anolis carolinensis* [222,223], a species with a 160 Myr-old sex chromosome system [212]. However, a lack of dosage balance under male heterogamety was found in *Basiliscus vittatus* and *Lialis burtonis* [224–226]. Rates of evolution in Z-linked genes were demonstrated to be increased, relative to their autosomal homologues in snakes, supporting the fast-Z effect [220]. Nevertheless, many questions remain regarding SD, dosage compensation and evolutionary rates of sex-linked loci, including the reasons for differences in the variability of SD among squamate lineages.

#### 

Testudines (turtles)

Despite their derived anatomy, turtles, containing 361 extant species [194], are related to the bird-crocodilian (Archosaurian) lineage, from which they split between the Upper Permian and Triassic, approximately 270–250 Ma [227], or earlier, in the Carboniferous, 320 Ma [228]. Twenty-two species have draft genomes assembled [42], specifically 18 Cryptodira and four Pleurodira (table 1). Turtles exhibit highly homologous and similarly sized genomes as crocodiles and some birds [229], ranging from 2 to 2.9 Gb [43]. Turtles are exclusively oviparous [197]. They comprise ESD (TSD) or GSD species, the latter with either ZZ/ZW or XX/XY systems [204,230–233]. While ESD is possibly ancestral to turtles and has been found in most studied species, GSD evolved independently at least five times and stayed notably stable in trionychids (ZZ/ZW) and probably also in chelids for many millions of years [233–235], although in chelids their XX/XY sex chromosomes display considerable morphological evolution, including a Y-to-autosome fusion [236]. No global dosage compensation was found in the female-heterogametic trionychid *Apalone ferox* [237], yet, dosage compensation varying by tissue, age, and temperature is suggested in *Apalone spinifera* [238]. Preliminary analyses of few sex-linked genes hint to fast-Z and slow-X effects in turtles [239,240]. Despite efforts to elucidate the molecular basis of GSD in turtles by searching for reptilian homologues of genes [232] involved in sexual development of mammals [241] and birds [242], no master SD gene has been identified yet [204]. However, *Sf1* (a testis development gene) is translocated to the ZW-chromosomes in *Apalone* and remains a candidate [243]. Natural polyploids are found in *Platemys platycephala*, specifically triploids, diploid–triploid mosaic and triploid–tetraploid mosaicism [54]. Transcriptomic analyses in turtles with ESD targeted the network of gonadal development [244–246], including its epigenetic regulation [246,247]. In early embryos of *Trachemys scripta*, the histone H3 lysine 27 (H3K27) demethylase *Kdm6b* has temperature-dependent sexually dimorphic expression. Knockdown of *Kdm6b* at 26°C (all-male offspring) triggers male-to-female sex reversal in more than 80% of embryos. *Kdm6b* directly promotes transcription of *Dmrt1* by eliminating the trimethylation of H3K27 near its promoter. Additionally, overexpression of *Dmrt1* was sufficient to rescue the sex reversal induced by disruption of *Kdm6b* [248]. Recent research revealed that temperature-mediated influx of calcium at 31°C drives phosphorylation of *Stat3*, which represses transcription of *Kdm6b* [249]. Still, many research questions on the genomics and molecular mechanisms of SD remain unanswered.

#### 

Archosauria (archosaurs)

*Crocodilia (crocodiles)* Crocodiles, containing only 24 extant species [194] diverged from birds more than 240 Ma [250,251], whereas forms, morphologically similar to the living crocodilians (Alligatoridae, Crocodylidae, Gavialidae), first appear in the fossil record 80–90 Ma [252]. With moderately large genome sizes (2.3–2.9 Gb; [251]), four genomes (*Alligator mississippiensis, Alligator sinensis, Crocodylus porosus, Gavialis gangeticus*) have been sequenced [251,253]. All crocodiles are oviparous [254]. Crocodiles have no sex chromosomes [255], and sexual differentiation is determined during development by a temperature-sensing mechanism with a poorly understood molecular basis. Earlier gene expression studies [256,257] have more recently been extended using gonadal RNAseq and revealed 41 differentially expressed/spliced genes at a male-producing temperature, including *Wnt1*, *Kdm6b*, *C/EBP* [258] and *Jumonji* chromatin modifiers [259]. In the Chinese alligator, orthologues of male-determining genes show an increasing or steady expression during gonadogenesis under the male-inducing but a decreasing expression pattern under the female-inducing temperature [260].

*Aves (birds)* Birds contain more than 10 000 extant species [261]. They shared the last common ancestor with the sister taxon of crocodiles earlier than 240 Ma [251,252]. Eased by high synteny [262] and compact genome sizes (0.9–2.1 Gb; [43]), over 502 [42] of bird genome assemblies have been published [263] and more are in preparation (table 1). Birds share homologous female-heterogametic sex chromosomes, i.e. a ZZ/ZW system [264]. No candidate for a female (W-specific) SD gene has been identified [265,266] and current knowledge strongly suggests that SD in birds is based on copy-number (i.e. dosage) variation of the Z-linked master SD gene *Dmrt1* with a key role in testis development, which is missing on the W [267]. The gene *Dmrt1* resides in the oldest evolutionary stratum of the Z-chromosome [268], shared by palaeognath and neognath birds [269–271]. A recent study using a CRISPR-Cas9 based mono-allelic targeting approach with sterile surrogate chicken hosts supports this hypothesis [272]. Such a chromosomally male (ZZ) chicken with a single functional copy of *Dmrt1* developed ovaries with typical female markers and exhibited follicular development. Interestingly, these animals were indistinguishable in external appearance from wild-type adult males, supporting that the development of male secondary sexual characters is driven by cell-autonomous sex identity and independent of gonadal hormones [272,273]. The rarity of Z0 and ZZW individuals in birds may suggest that these genotypes are often lethal or infertile [274], and that a locus on the W might control dosage compensation of some Z-linked genes [275,276]. Lethality of polyploid bird embryos may be owing to a general disruption of development. Mortality was high among ZZZ individuals, which developed as males [277]. In a study of 4182 chicken embryos, haploids (1.4%), triploids (0.8%, 9 ZZZ, 7 ZZW, 15 ZWW) and tetraploids (0.1%, 1 ZZZZ, 1 ZZWW) were found, none of which survived to hatching ([278]; discussed in: [279]). ZZ-eggs can be sex-reversed to female by oestrogen-exposure during the critical period of gonad formation [7]. Gynandromorphs with male versus female bilateral morphology can arise from double fertilization of a binucleate egg and this bilaterally distinctive chromosomal constitution of cells governs perception of the hormone environment [7]. Facultative parthenogenesis in birds mostly leads to early embryonic mortality, but hatchlings or even adults (all males) were reported in turkey and chicken [52,53]. Multiple neo-sex chromosomes have been found only extremely rarely in birds [280]; however, extended Z and W chromosomes, formed by addition of autosomal material to both Z and W chromosomes, evolved within songbirds in the Sylvioidea superfamily [281–283] and in *Eopsaltria australis* [284].

Genomics of avian sex chromosomes is well studied and revealed great interspecies diversity of pseudoautosomal regions (PAR) and Z/W differentiation, from relatively modest degradation in some palaeognath species to extreme degradation in most modern birds [285,286]. The PAR is short in many neognaths, and even without genes in chicken [287]. Similar to the surviving genes on the mammalian Y chromosomes, the retained genes on the bird W chromosomes are enriched for housekeeping or putative dosage-sensitive genes with stronger selective constraints than the lost ones, and are conserved between distantly related lineages of birds [287,288]. Shared or lineage-specific recombination suppression produced ‘evolutionary strata’, i.e. punctuated sequence divergence owing to stepwise suppression of recombination between Z and W [268]. These strata evolved by a complex process of W- and Z-linked inversions, the latter comprising 25 in total across avian lineages [270].

All studied birds exhibit incomplete ZZ/ZW dosage compensation [289], which seems gene-specific and partial [290]. Moderation of expression levels partially balances out the otherwise twofold difference [291,292], presumably because not all genes are equally sensitive to dosage differences. For many genes, this twofold expression difference does not appear to be associated with severe fitness costs. In addition, other bird genes have evolved sex-biased expression [285,293]. Likewise, in palaeognath birds, sex chromosome genomics recently revealed incomplete dosage compensation, confirmed large (more than 100 Myr-old) PARs, where genes in some species, however, evolve faster than autosomal ones [294]. Like other sex chromosomes, those of birds accumulate transposable elements in the non-recombining regions of the W [295]. On the W, Peona *et al.* [296] revealed enrichment of endogenous retroviruses, which can be expressed and may retrotranspose, inducing genome-wide female-biased mutation rates. Furthermore, probably all songbirds have a germline-restricted chromosome (GRC) and thus undergo a form of partial genome elimination [40,41]. First cytogenetically described in zebra finch, *Taeniopygia guttata* [297], GRC is absent in somatic cells but present in one copy in male germline cells (but eliminated during spermatogenesis) and two copies in female germline cells (reviewed in [298,299]). Recent genomic, transcriptomic and comparative cytogenetic work suggests that the GRC is enriched in genes [300–302]. The zebra finch GRC contains more than 115 paralogues to single-copy genes on 18 autosomes and the Z is enriched in genes involved in female gonadal development. These genes are transcribed in testes and ovaries [301]. Although the exact function of GRC is currently unclear, the GRC resembles an XX/X0 system, albeit one limited to the germline on top of a ZZ/ZW system in germline and soma. Another level of complexity for understanding the songbird *sexome* arises from the proposed maternal inheritance of the GRC (but see [303]), implying that it is co-inherited with the W and the mitochondrial genome.

#### Mammalia (mammals)

Monotremata (monotremes) With five extant species [137], this order includes the sole representatives of the subclass Prototheria, which diverged 200 Ma from viviparous mammals (Theria; [304]), represented by Ornithorhynchidae with a single species (platypus) and the 50 Ma diverged Tachyglossidae (echidnas) with four species. Platypus and echidna genomes are among the smallest in mammals (2.7–2.8 Gb; [43]). Monotremes display a fascinating mixture of derived mammalian and primitive amniote morphological and physiological features shared with sauropsids (reptiles including birds), and have a unique reproductive system that combines egg-laying with lactation. Likewise, the platypus genome exhibits a combination of derived and plesiomorphic characters [305]. The echidna genome has just become available [306]. The monotreme karyotypes have been controversial for almost half a century (cf*.* [307]) but turned out to contain multiple sex chromosomes, which probably arose from sequential rearrangements between ancient sex chromo­somes and several autosomes. During gametogenesis, meiotic chains form that comprise 10 sex chromosomes (five Xs and five Ys) in male platypus and 9 (five Xs and four Ys) in male echidnas [307,308]. This monotreme sex chromosome system evolved independently of the sex chromosomes of viviparous mammals approximately 175 Ma [304,309]. The mammalian master SD gene, *Sry*, is absent from the genome, while the putative avian SD gene, *Dmrt1*, is located on the chromosome X_5_, in two copies in females and one in males, i.e. the opposite situation from birds [310]. The most promising master SD candidate is *Amh* (*AmhY*), which is known to have a fundamental role in SD of fishes, and is carried by the Y_5_ chromosome that corresponds to the oldest of the evolutionary strata of the monotreme sex chromosomes [304,311]. The recent improvement of a male platypus genome revealed seven strata, distributed across the five Xs, which sequentially suppressed recombination with their homologous Ys, five of which are shared with echidna [306]. This work also provided insights into the origin and evolution of the 10 platypus sex chromosomes. Sequence homology was found between the chromosome Y_5_, where *AmhY* is located, and the chromosome X_1_, suggesting that the 10 platypus sex chromosomes ancestrally formed a ring, rather than a chain. In contrast to autosomes, there are extensive interchromosomal contacts between the extant platypus sex chromosome pairs. Unusually frequent interchromosomal contacts were also found between the autosomal regions in humans homologous to the platypus sex chromosomes, suggesting that reciprocal translocations leading to the evolution of the multiple platypus sex chromosomes were facilitated by spatial proximity of these chromosomes that pre-existed in the mammalian ancestor. Monotreme dosage compensation of X-linked genes occurs on a gene-by-gene basis [312], rather than through chromosome-wide silencing, as in eutherians and marsupials [313,314].

### 

Theria (viviparous mammals)

*Metatheria (marsupials)* Marsupials diverged approximately 180 Ma from Eutheria (placentals) [304] and contain 385 extant species [136,137], inhabiting Australasia and the Americas. Marsupials exhibit moderate genome sizes of approximately 3.9 Gb [43,315]. To date, eight genomes have been sequenced [42] and genomic evolution has recently been reviewed [316]. Marsupials differ from eutherian mammals in many features of reproduction and development, e.g. extraembryonic tissues have undergone remarkable modifications to accommodate reduced egg size and quantity of yolk/deutoplasm versus increasing emphasis on viviparity and placentation [317]. While all marsupials show male heterogamety (XX/XY), the X of marsupials vary substantially in size, morphology and banding patterns, even between species with an ancestral-like 2*n* = 14 karyotype [318]. The marsupial X shares complete homology with two-thirds of the eutherian X, the remaining third is autosomal in marsupials and corresponds to an early addition on the eutherian lineage. The marsupial X, therefore, represents the ancestral therian X [285]. Translocations or fusions between autosomes and sex chromosomes have been observed in several marsupials [207,319]. While marsupials usually inactivate the paternal X chromosome in the female soma by a marsupial-specific non-coding RNA (RSX: RNA on silent X; [320]), dosage compensation often remains incomplete, contrasting to random but tightly controlled eutherian X inactivation [321]. Marsupial dosage compensation is associated with specific epigenetic modifications [322]. Cytogenetics in some bandicoots (family Peramelidae) revealed somatic elimination of one X in females and the Y in males at different ontogenetic stages, resulting in sex chromosome mosaics in various tissues [323]. The marsupial Y is much smaller than the eutherian Y; marsupial X and Y do not share a PAR, and thus cannot form a synaptonemal complex or recombine during the first meiotic division, but a special structure, the dense plate, maintains sex chromosome association to ensure proper segregation [319,324,325]. Marsupial Y chromosomes share the master male SD gene, *Sry*, with placental Y chromosomes [316,326].

*Eutheria (placentals)* Placental mammals diverged 180 Ma from marsupials [304] and with 6992 species comprise the vast majority of living mammals [136,137]. Genome size varies between approximately 2.7 Gb in Laurasiatheria, approximately 3.3 Gb in Supraprimates/Euarchontoglires, approximately 4.4 Gb in Xenarthra and approximately 5.3 Gb in Afrotheria [315], with the largest mammalian genome (approx. 7.7 Gb) being that of a rodent from South America, *Tympanoctomys barrerae* [327]. Placental genome assemblies are available from 411 species [42] (table 1). Presumably owing to sex-specific methylation [328] and/or other aspects of development [279], no polyploid mammals are viable and reports on natural polyploids have been disproved [327,329]. Eutherian sex chromosomes evolved from a pair of autosomes in the therian lineage around 180 Ma, they are nowadays highly differentiated in both size and gene content owing to the arrest of recombination causing the degeneration of the Y [285,330]. The eutherian X chromosome carries more than 1000 genes, whereas the Y contains only a few protein-coding genes [304]. The degree of heteromorphism and PAR of eutherian sex chromosomes can differ dramatically, e.g. humans exhibit two PARs with the larger of about 2.5 Mb, whereas the house mouse PAR is only 0.5 Mb, and other species have even lost their PAR [331,332]. Lineages with multiple neo-sex chromosomes (X_1_X_2_X_1_X_2_/X_1_X_2_Y or XX/XY_1_Y_2_) have independently evolved by fusion with an autosome at least 20 times [207]. In some rare cases, a translocation of an autosome to both sex chromosomes has restored a large segment of homology between X and Y, creating a neo-PAR, as found in the African pygmy mouse (*Mus minutoides*), where it appears to show signs of early stages of sex chromosome differentiation [333]. Eutherians randomly inactivate one of two Xs in female somatic cells by a non-coding RNA (*Xist: X-inactive specific transcript*; [334]). Active and inactive X chromosomes localize to different subnuclear positions with distinct chromosomal architectures and epigenetic signatures, reflecting their activity state [335]. The eutherian Y exhibits strata that stopped recombining at well-dated time points [304] and carries the master SD gene (*Sry*). This testis development initiating transcription factor is homologous to the X-linked *Sox3* [7,336]. The gene regulatory network of male and female SD- and developmental pathways are best-studied in laboratory house mice [7]. While for 30 years *Sry* has been thought to comprise a single exon, a cryptic second exon, essential for male SD in mice has just been identified [337]. Although eutherian XX/XY sex determination is extremely conserved, a few rodent species evolved unusual, derived sex chromosome systems [338]. For example, spiny rats, *Tokudaia osimensis*, and mole voles, *Ellobius lutescens*, have lost their Y chromosomes including *Sry* [339,340], and the gene *etv* is hypothesized to activate *Sox9* [7]. On the other hand, fertile females with a Y chromosome are known in some rodents (e.g. *Akodon azarae*). The situation is probably best explored in the African pygmy mouse (*Mus minutoides*), with a sex reversal mutation on a mutant X (called X*) and only XY individuals presenting phenotypic males, while genotypic XX, XX* and X*Y mice are females [341]. Genotypic XX females in moles (*Talpa occidentalis*) develop ovotestes instead of ovaries and exhibit a masculinized phenotype (musculature, external genitalia, aggressiveness). The testicular part of the ovotestes lacks fertile germ cells but contains typical male androgen-producing cells. Recently, it was uncovered that the increased androgen synthesis in female moles is caused by a tandem triplication of a region containing *Cyp17A1*, a gene controlling androgen synthesis, and an intrachromosomal inversion involving the pro-testicular growth factor gene *Fgf9*, heterochronically expressed in the ovotestes [342]. Adult mammals cannot perform sex reversal but genetic perturbations can destabilize the commitment to Sertoli and granulosa cell fate in adult life [7], showing that adult mammalian testes or ovaries require repression of the alternative state [343,344]. Gene expression in eutherians across 12 tissues (human, macaque, mouse, rat, dog) revealed hundreds of genes with conserved sex-biased expression but showed that it has arisen recently and is thus not shared between most mammals [345]. XX-genotypes have been experimentally shown to increase lifespan in mice [346].

## 3. Beyond whole vertebrate genomes: a pledge for ‘*sexomics’*

There are several ongoing initiatives to sequence many of the 71 000 vertebrate genomes [347–350]. In context to future research on vertebrate SD and differentiation, we hereby suggest that future sequencing efforts target species with missing information on their SD system, the sex chromosomes or special developmental and/or reproductive modes of interest. As an overview, we have prepared table 1, a summary of the electronic supplementary material, table S1, which summarizes currently (December 2020) available whole-genome information in the context of knowledge on sex evolution from [42]. This is where we are now and we think that sequencing technology and bioinformatics will make it increasingly easier to obtain high-quality genomes from non-model species.

**Figure 2.**
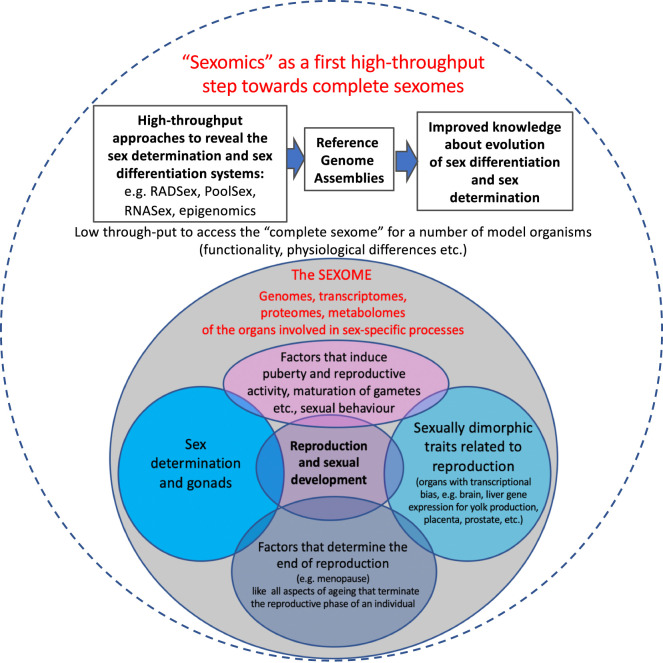
Scheme on *sexomics* as a first high-throughput step to improve our understanding of the complete *sexome* of vertebrates.

An obvious priority for the *sexome* (figure 2) to be examined by *sexomics* is the sequencing and assembly of sex chromosomes in taxa possessing them. Assembling the sex-limited sex chromosome, the Y or W, has been historically difficult owing to the accumulation of repetitive elements and palindromic sequences on the Y and W [287,351]. Many early genome assembly projects chose to sequence the homogametic sex (XX or ZZ individuals) to avoid problems with assembling sex chromosomes and prevent mis-assembly [352,353]. The advent of long-read sequencing, e.g. PacBio and Oxford Nanopore, has made assembly of the hemizygous sex chromosome (Y or W) feasible, and many genome assembly consortia are now using the heterogametic sex as the reference assembly [354,355]. However, *sexomics* is more than just including sex chromosomes in genome assemblies.

While genome sequencing *per se* will undoubtedly present a driving force towards our understanding of vertebrate sex, we wish to point out that genome sequencing is only a starting point to comprehensively understand SD and sex evolution. For integrative research from here and far beyond, we propose to introduce the terms *sexome* (and *sexomics*; figure 2). As the *sexome*, we consider the information about an individual regarding its sexual differentiation, development and reproduction on all levels of biological organization. This includes the genomic and epigenetic information, the transcriptomes of the organs involved in these sex-specific processes. These organs comprise the gonads, secondary sex organs and characters, organs with a transcriptional sex-bias (e.g. brain, liver gene expression for yolk production, placenta, prostate), the respective proteomes as well as information about environmental factors that induce puberty and reproductive activity, maturation of gametes, etc., sexual behaviour, and finally the factors that determine fertility and the end of or transitions in reproduction (e.g. menopause). It should also include information on malfunction and impairment (e.g. teratology and endocrine disruption).

While we will not be able to cover this universe, we first focus on the analyses of genomes, transcriptomes and proteomes and how they influence the whole picture. We also would like to encourage others (neurobiologists, ethologists, ecologists) to contribute their expertise to complete the *sexome* (figure 2) of as many species as possible.

Like other ‘-*omics*’ terms, *sexomics* describes a special feature of an organism, and the *sexomics* idea is a term to gather all relevant ‘-*omics*’ approaches, applicable in high-throughput mode. We argue that the *sexome* in the first place is a comprehensive description, which comprises all aspects of sexual development and is an archive of data that characterizes a complex phenotype, specific to the reproductive mode of an organism (e.g. female, male, hermaphrodite). Information about the *sexome* feeds into the classical disciplines (see above) and should be considered at the level of ‘comparative *sexomics*' as a tool for improving the approaches to a better understanding of molecular and phenotypic evolution, population dynamics, ecology and more. We are convinced that only such comparative approaches across the phylogeny as well as information on intraspecific and intra-population variation, and its regulation will lead to substantial scientific progress. We are sure that this holds particularly true for the *sexome*.

Elucidating the evolution of sex chromosomes and SD in non-model vertebrates primarily addresses fundamental research questions [2,11], including turnovers of SD systems [356], speciation [357], hybridization [358] and evolutionary development [359]. Likewise, based on similarities and differences in SD and sexual differentiation in non-vertebrates, such as insects and other arthropods, genomic and molecular links between these major taxonomic groups (e.g. the role of the *Dmrt* gene family [360]) may allow us to consider *sexomics*-like approaches in other organismal groups.

Beyond basic research, we emphasize that integrative *sexomics* research in vertebrates will also be of high relevance for many fields of applied research. For example, a major, still poorly understood and complex threat for aquatic and semi-terrestrial vertebrates is endocrine disruption [361]. Missing knowledge on the developmental biology of sex and the genetics of SD remains a major obstacle to study endocrine-disruptive effects in many non-model fishes and amphibians [175,362], and such applications would improve bioindication in freshwater bodies to sense threats for humans. A second field, with relevance to better protect wild fish populations, is sustainable aquaculture with an increasing demand to control sex ratios or to foster mono-sex production [363]. We hope that our review, which does not claim to be complete, will provide a stimulus for upcoming and future research.

## Data accessibility

This article has no additional data.

## Glossary

*Autosomes:*: all chromosomes of the nucleus, which are not sex chromosomes (or B chromosomes).
*Budding:*: a type of asexual reproduction, where the new organism emerges gradually as a bud from the cellular membrane or a body part of the parent.
*Deduplication*: the process by which duplicated genes revert to single-copy genes owing to loss or silencing of one copy.
*Diploidization*: the process by which a polyploid genome turns functionally and structurally (e.g. by gene expression changes, chromosome loss or divergence between homologous chromosomes in autopolyploids) into a diploid state.
*Dosage balance:*: (also ‘male-to-female expression balance’, or ‘parity in the expression between sexes’): the molecular mechanisms that equalize the expression of X-/Z-linked genes missing copies on the Y/W chromosomes between sexes, regardless of whether the ancestral expression levels are restored.
*Dosage compensation*: the molecular mechanisms that restore the expression of X-/Z-linked genes with missing copies on the Y/W chromosome equalizing the expression of these genes between sexes, according to some definitions, to the ancestral expression levels.
*Environmental sex determination*: sex is determined by environmental factors, most commonly temperature (TSD) during a sensitive embryonic stage of gonadal development, in species without sex-specific sequence differences in their genome.
*Epigenetic modification*: reversible changes that modify the genetic material and regulate expression without affecting the DNA sequence (e.g. DNA methylation, histone modification).
*Evolutionary stratum*: a region of the sex chromosomes, which ceased recombination in a single step and thus evolutionary period (plural: ‘evolutionary strata’).
*Exotrophic larvae*: larvae feeding on external resources (including maternal trophic eggs) (opposite term ‘endotrophic’).
*Facultative parthenogenesis*: occasional parthenogenetic reproduction in a species that typically reproduces sexually.
*Fast(er)-X/Z effect*: the accelerated evolutionary rate of X- or Z-linked sequences relative to that of autosomal loci.
*Female heterogamety*: species with sex determination controlled by a ZZ/ZW sex chromosome system (and similar derived systems, e.g. with multiple sex chromosomes).
*Gametogenesis*: the developmental process to produce (usually sex-specific and usually haploid) cells specialized for reproduction (gametes), commonly referred to as ovum/egg (in females) and sperm (in males).
*Gametologues*: homologous genes shared by sex chromosomes (e.g. between X and Y chromosomes) in their non-recombining parts.
*Genome elimination*: regulated loss of genomic regions during development of an organism.
*Genotypic sex determination (GSD)*: sex is determined by a sex-specific genomic region (at least a single-nucleotide polymorphism), most commonly by a sex-specific combination of chromosomes (i.e. sex chromosomes).
*Germline-restricted chromosomes (GRCs)*: type of partial genome elimination where one or more entire chromosomes, the GRCs, are lost during germline-soma differentiation.
*Gonochorism*: having just one of at least two distinct sexes in any one individual organism throughout its lifetime.
*Gynogenesis*: reproductive mode, requiring the activation of embryogenesis by sperm without contribution of paternal DNA (often also called ‘sperm-dependent parthenogenesis' or sometimes ‘pseudogamy’).
*Hemizygous genes*: genes present in a single copy in an otherwise diploid cell, typically Y- and W-specific genes, and likewise X-specific genes in males (i.e. genes present on the X and missing on the Y chromosome or X-linked genes in X0-males of XX/X0 systems), and Z-specific genes in females.
*Hermaphroditism*: developing both male and female gametes during the life cycle of an organism.
*Heterogametic sex*: the sex that produces two types of gametes that each contain one of two different types of sex chromosomes, e.g. XY-males or ZW-females.
*Heteromorphic sex chromosomes*: sex chromosomes that are morphologically distinguishable when viewed with a light microscope (opposite term: ‘homomorphic’).
*Holoblastic (total) embryonal cleavage*: the zygote and blastomeres are completely divided during the cleavage.
*Homeologues*: orthologous genes derived from different lineages or species, combined by hybridization in the same diploid or polyploid genome.
*Homeologous chromosomes*: homologous chromosomes derived from different lineages or species, combined in the same genome of hybrid origin (diploid or polyploid).
*Homeothermic*: having stable, usually physiologically maintained, internal temperature.
*Homogametic sex*: the sex that produces gametes that all contain the same type of sex chromosomes, e.g. XX females or ZZ males.
*Homomorphic sex chromosomes*: sex chromosomes that are morphologically indistinguishable in size and shape (opposite term: ‘heteromorphic’).
*Hybridogenesis*: reproductive mode with selective (usually clonal: ‘hemiclonal’, i.e. ‘half clonal’) transmission of one of the two parental genomes of hybrids to their offspring; more complex inheritance patterns may occur in hybrid polyploid organisms, sometimes collectively termed ‘hybridogenesis’ (or ‘meroclonal’, i.e. ‘partly clonal’, inheritance).
*Introgression*: the transfer/moving of genetic/genomic material from one population or species into the gene pool of another, by hybridization.
*Kleptogenesis*: reproductive mode of a hybrid unisexual species, requiring sperm from a related, often parental, species to trigger the embryonic development; the sperm can either be eliminated (see Gynogenesis) or its genome can be partially or completely incorporated.
*Male heterogamety*: species with sex determination controlled by an XX/XY sex chromosome system (and similar derived systems, e.g. with multiple sex chromosomes).
*Menopause*: post-reproductive life period after the end of female reproduction; known from few cetaceans and hominins.
*Meroblastic (partial) embryonal cleavage*: the cleavage furrows do not completely divide the fertilized egg (usually in eggs with large amounts of yolk).
*Mosaicism*: the presence of more than one population of somatic or germline cells with different genotypes or ploidies within an individual.
*Neo-sex chromosome*: derived sex chromosome, formed by fusion of the ancestral sex chromosome with an autosome.
*Neoteny*: reproduction while retaining juvenile characteristics, e.g. in amphibians (urodela) without full metamorphosis.
*Obligate parthenogenesis*: offspring are produced exclusively by parthenogenesis.
*Ohnologues*: paralogous genes originated from whole-genome duplications, in vertebrate research usually understood as diversified paralogues, evolved by ancient whole-genome duplications.
*Orthologues*: homologous genes originated from a single common ancestor, now present in different genomes (usually separated by a speciation event).
*Oviparity*: the egg is expelled and the embryo largely develops and hatches outside the body of the mother.
*Ovo-viviparity*: the egg develops until hatching within the mother, where the embryo is feeding exclusively on nutrients pre-deposited in the egg.
*Ovotestis*: a gonad with both ovarian and testicular tissues (irrespective of their functionality).
*Palindromic sequence*: complementary short DNA or RNA sequence motifs, arranged in close proximity but with opposite orientation; they can potentially form secondary structures, such as hairpins.
*Parthenogenesis*: reproductive mode by which offspring (or at least an embryo) is produced from an egg without genetic contribution from sperm.
*Paralogues*: homologous genes in the same genome, originated by duplication (local gene duplication, whole-genome duplication).
*Poikilothermic*: having variable, usually environmentally dependent, internal temperature.
*Ploidy*: the number of the complete sets of chromosomes in a eukaryotic cell (i.e. one set = haploidy, two sets = diploidy).
*Polygenic sex determination*: sex is controlled by multiple genes (also called ‘multilocus sex determination’).
*Polyploidy*: more than two complete sets of chromosomes occur in a eukaryotic cell.
*Protandrous hermaphroditism*: producing male gametes in the first stage of the life cycle, and female gametes in a later stage of an organism.
*Protogynous hermaphroditism*: producing female gametes in the first stage of the life cycle, and male gametes in a later stage of an organism.
*Pseudoautosomal region*: recombining part of sex chromosomes.
*Recombination*: the exchange of genetic material between homologous chromosomes, occurring during meiosis (most frequent and regularly) or mitosis in eukaryotes.
*Sequential hermaphroditism*: producing female and male gametes at different periods of the life cycle of an organism.
*Sex determination*: the developmental process deciding the sex of the individual.
*Sex-determining locus*: a locus determining the sex of an individual, in vertebrates triggering the differentiation of the initially bipotential gonad either towards testis or ovary.
*Sex chromosomes*: chromosomes that carry a sex-determining locus (or loci) and segregate in a sex-specific manner.
*Sex chromosome differentiation*: the process leading to changes in content and structure between the homologous X and Y (or Z and W) sex chromosomes, involving one or more of the following events: accumulation of sexually antagonistic alleles, loss of functional genes, recombination arrest, chromosomal rearrangements, heterochromatinization and/or accumulation of repetitive elements.
*Sex chromosome turnover*: evolutionary switch from one sex chromosome system to another, e.g. by the emergence of new master SD genes on new chromosomes or translocation of the sex-determining locus to another chromosome.
*Sex reversal*: the change of sex during the development of an organism, evident by a mismatch between gonadal phenotype (phenotypic sex) and sex-specific genotype (genotypic sex).
*Simultaneous hermaphroditism*: producing both female and male gametes at the same time in one organism.
*Synaptonemal complex*: a protein structure that connects paired homologous chromosomes during the meiotic prophase in eukaryotes.
*Transcription factor*: protein that regulates the transcription of genes.
*Transposable element*: genetic elements capable of mobilizing via copy-and-paste (retrotransposons, including endogenous retroviruses) or cut-and-paste mechanisms (most DNA transposons).
*Viviparity*: the egg develops exclusively within the mother, where the embryo is feeding on nutrients, regularly provided by the mother.
